# Supplementary material for: Serendipity and strategy in rapid innovation
Source: Nat Commun. 2017 Dec 8;8:2002. doi: 10.1038/s41467-017-02042-w (PMC5722871; doi:10.1038/s41467-017-02042-w)
Supplement: Supplementary file 1 — Supplementary Information [file 41467_2017_2042_MOESM1_ESM.pdf]

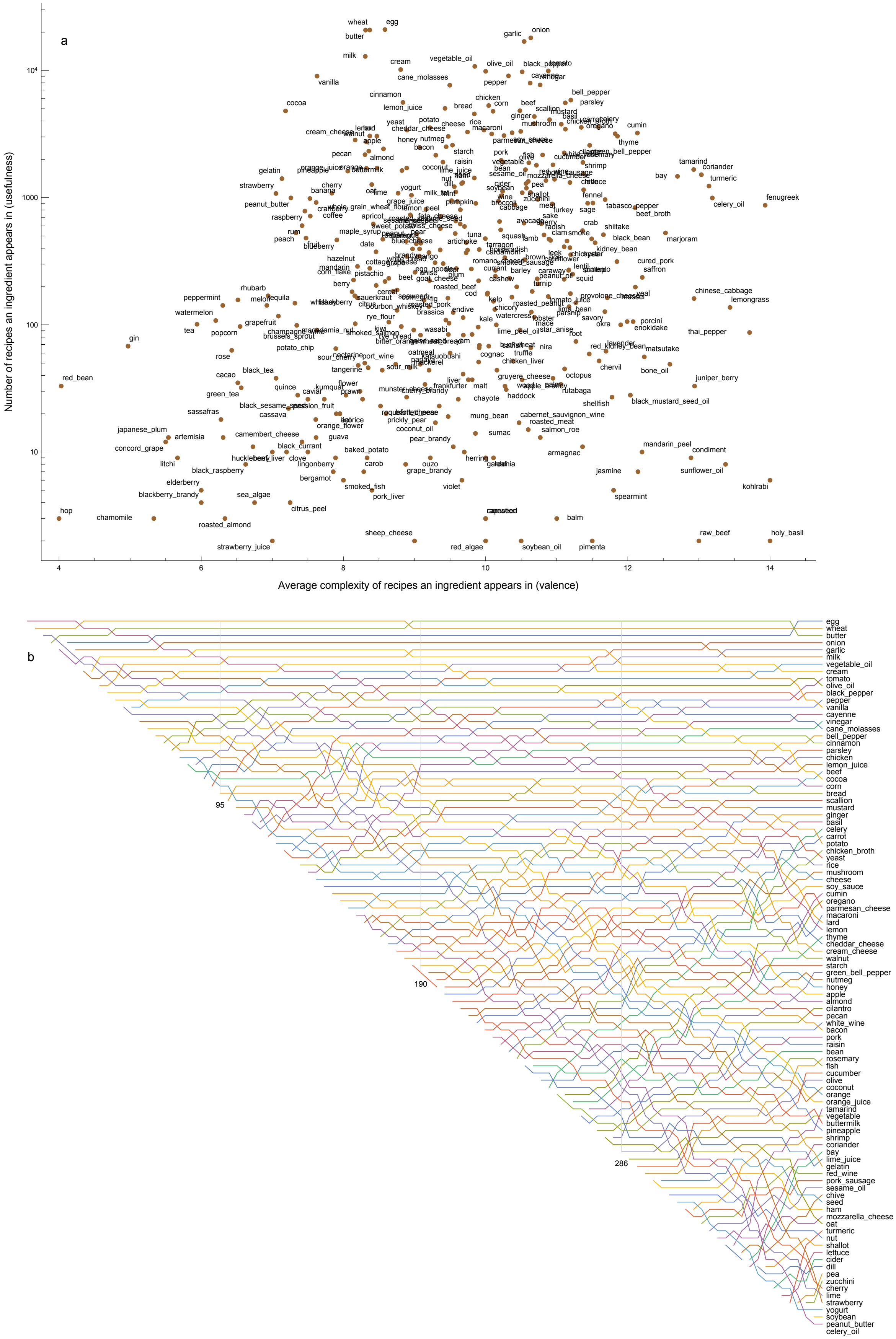

Supplementary Figure 1: The properties of ingredients in gastronomy imply changes in their relative usefulness over time. **a** The valence-usefulness scatter plot at stage N for all ingredients that are used in two or more recipes (365 of the 381 ingredients). **b** The relative usefulness of different ingredients as the number of ingredients we possess increases, for the 100 ingredients most useful when we have all 381 ingredients.

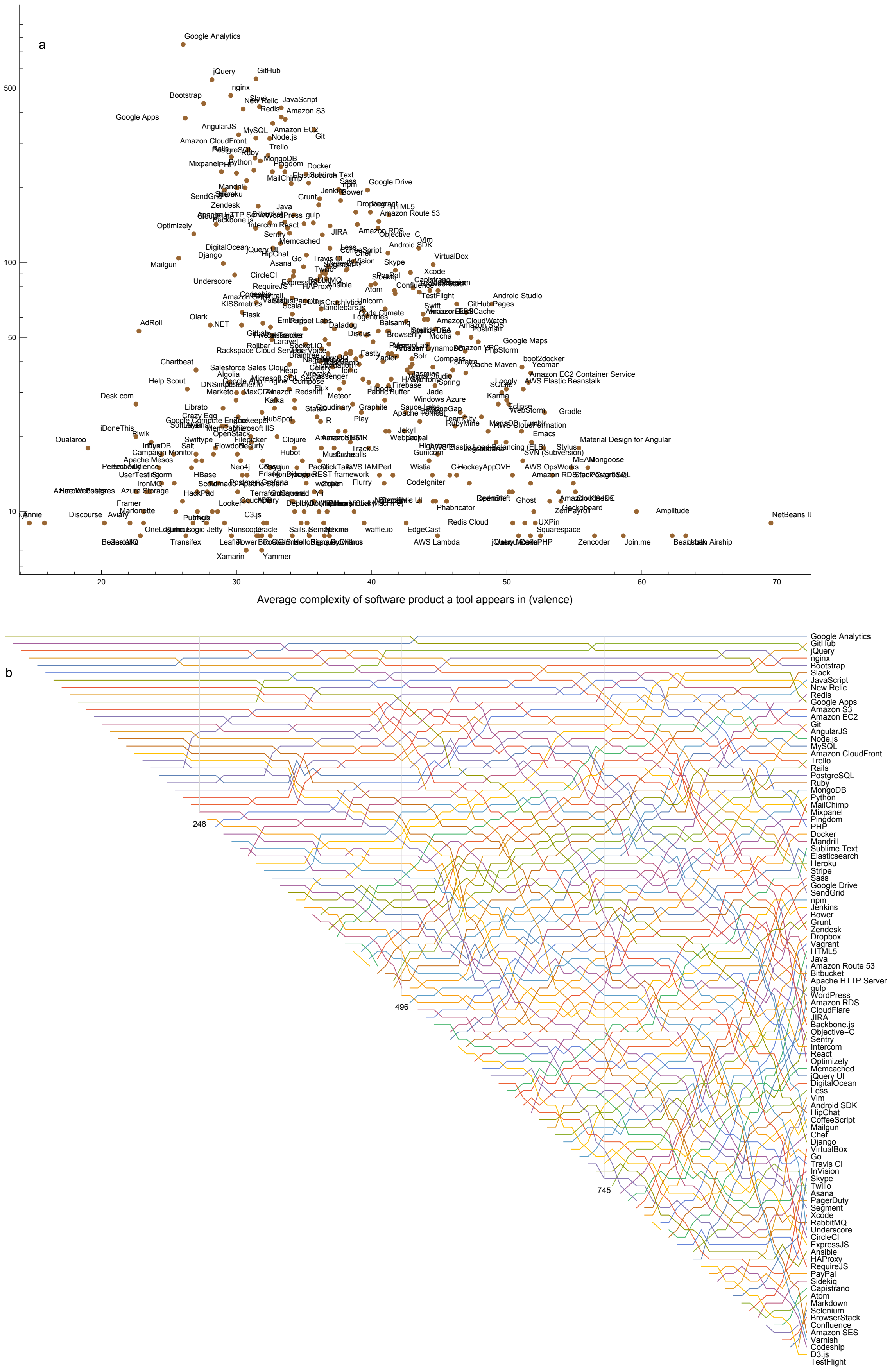

Supplementary Figure 2: The properties of technology components imply changes in their relative usefulness over time. **a** The valence-usefulness scatter plot at stage N for the 365 technology tools most useful in making software products. **b** The relative usefulness of different tools as the number of tools we possess increases, for the 100 tools most useful when we have all 993 tools.
